# Management of gout in chronic kidney disease: a G-CAN Consensus Statement on the research priorities

**DOI:** 10.1038/s41584-021-00657-4

**Published:** 2021-07-30

**Authors:** Lisa K. Stamp, Hamish Farquhar, Huai Leng Pisaniello, Ana B. Vargas-Santos, Mark Fisher, David B. Mount, Hyon K. Choi, Robert Terkeltaub, Catherine L. Hill, Angelo L. Gaffo

**Affiliations:** 1 University of Otago, Christchurch, New Zealand; 2 Discipline of Medicine, Faculty of Health and Medical Sciences, University of Adelaide, Adelaide, South Australia Australia; 3 Department of Internal Medicine, Rio de Janeiro State University, Rio de Janeiro, Brazil; 4 Harvard Medical School and Massachusetts General Hospital, Boston, MA USA; 5 Prima CARE, Fall River, MA USA; 6 Renal Divisions, Brigham and Women’s Hospital, Boston, MA USA; 7 VA Boston Healthcare System, Boston, MA USA; 8 Division of Rheumatology, Allergy, and Immunology, Department of Medicine, Massachusetts General Hospital, Boston, MA USA; 9 VA San Diego Healthcare System, San Diego, CA USA; 10 Department of Medicine, University of California San Diego, La Jolla, CA USA; 11 Rheumatology Unit, The Queen Elizabeth Hospital, Woodville, South Australia Australia; 12 University of Alabama at Birmingham, Birmingham, AL USA; 13 Birmingham VA Medical Center, Birmingham, AL USA

**Keywords:** Gout, Medical research

## Abstract

Gout and chronic kidney disease (CKD) frequently coexist, but quality evidence to guide gout management in people with CKD is lacking. Use of urate-lowering therapy (ULT) in the context of advanced CKD varies greatly, and professional bodies have issued conflicting recommendations regarding the treatment of gout in people with concomitant CKD. As a result, confusion exists among medical professionals about the appropriate management of people with gout and CKD. This Consensus Statement from the Gout, Hyperuricemia and Crystal-Associated Disease Network (G-CAN) discusses the evidence and/or lack thereof for the management of gout in people with CKD and identifies key areas for research to address the challenges faced in the management of gout and CKD. These discussions, which address areas for research both in general as well as related to specific medications used to treat gout flares or as ULT, are supported by separately published G-CAN systematic literature reviews. This Consensus Statement is not intended as a guideline for the management of gout in CKD; rather, it analyses the available literature on the safety and efficacy of drugs used in gout management to identify important gaps in knowledge and associated areas for research.

## Abstract (web summary)

In this Consensus Statement, members of the Gout, Hyperuricemia and Crystal-Associated Disease Network (G-CAN) highlight gaps in knowledge about the management of gout in people with chronic kidney disease, and identify important areas for future research to address challenges in the treatment of this patient population.

## Introduction

Gout is the most common form of inflammatory arthritis in men over the age of 40 years. The prevalence of gout has been reported to range from 0.1% to 10%^1,2^. The prevalence of gout is generally higher in men (5.2%) than in women (2.7%) according to the most recent data from the US National Health and Nutrition Examination Survey (NHANES)^2^. Kidney impairment is common in people with gout: as many as ~70% of adults with gout have an estimated glomerular filtration rate (eGFR) of <60 ml/min/1.73 m^2^, and 20–24% have an eGFR of <30 ml/min/1.73 m^2^ (Table 1)^3,4^. Reduced GFR is a risk factor for the early development of tophi, suggesting that renal function might modulate the severity of gout^5,6^. The reverse is also true, as the prevalence of gout is higher in people with chronic kidney disease (CKD): 24% of adults with an eGFR of <60 ml/min/1.73 m^2^ have gout compared with 2.9% of adults with an eGFR of >90 ml/min/1.73 m^2^ (ref.^7^). The prevalence of gout is higher in men with CKD than in women with CKD^7^. Hyperuricaemia (defined as a serum urate level of >6.8 mg/dl in men and >6.0 mg/dl in women) is also common in the context of advanced CKD, with a prevalence of 64% in people with stage 3 CKD and 50% in those with stage 4 or 5 CKD^7^.

**Table 1:** Stages of CKD

Stage	Description	eGFR (ml/min/1.73 m^2^)
1	Normal or high GFR	≥90
2	Mild CKD	60–89
3A	Mild to moderate CKD	45–59
3B	Moderate to severe CKD	30–44
4	Severe CKD	15–29
5	End-stage CKD	<15

CKD, chronic kidney disease; eGFR, estimated glomerular filtration rate.

Monosodium urate (MSU) crystals, which form in the presence of hyperuricaemia, cause gout flares in large part by activating monocytes and macrophages, with resultant NLRP3 inflammasome-mediated IL-1β release, many other local and systemic high-grade pro-inflammatory responses, and articular neutrophil influx and activation^8^. Hyperuricaemia is an amplifying factor for MSU crystal-induced inflammation, priming certain monocyte–macrophage pro-inflammatory responses in humans and mice^9^. In this context, evidence from multiple studies supports a low-grade inflammatory phenotype in CKD, which is linked with increased serum concentrations of C-reactive protein, many pro-inflammatory cytokines, prostaglandins and leukotrienes, and with intestinal dysbiosis^10^. The crosstalk between the systemic inflammatory states of CKD and gout, as well as common comorbidities of both diseases that are modulated by low-grade inflammation, is likely to have clinical consequences. The same is the case for the inflammation-modulating effects in CKD and gout of obesity, type 2 diabetes mellitus and renin–angiotensin system activation in the pathophysiology of hypertension, and for the use of statins (which modulate trained immunity in monocytes and macrophages) and metformin and ω-3 fatty acids (which inhibit MSU crystal-induced inflammation)^11–13^. Additionally, ultrasonography studies have demonstrated renal medullary echogenicity in patients with severe gout^14^, potentially attributable to MSU crystalluria and the development of tophi within the renal medulla^15^. MSU crystal-driven inflammation might thus directly affect renal structure and function in patients with gout.

**Fig. 1:**
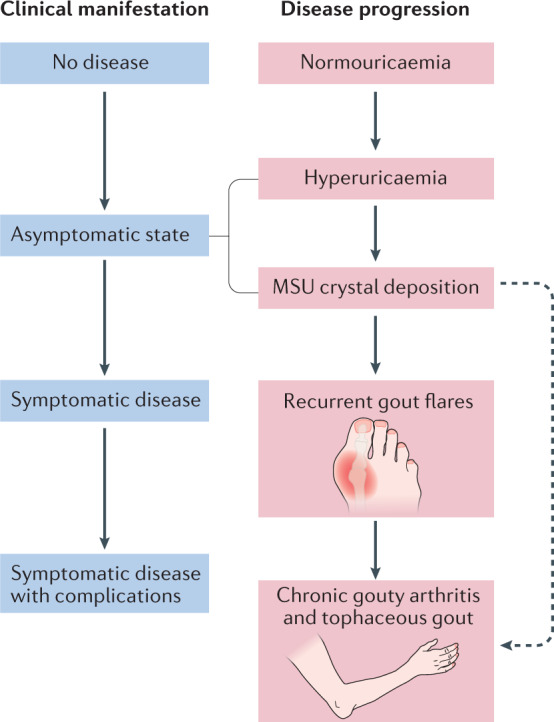
Stages of gout. Gout progresses through several classic disease stages and corresponding clinical manifestations. In some cases, advanced disease stages and complications can appear prematurely, without earlier disease stages or clinical manifestations being apparent (for example, tophaceous gout without prior gout flares), although this pattern is uncommon. MSU, monosodium urate. Adapted from ref.^80^, Springer Nature Limited.

There is a paucity of data on the natural history of gout, which has several stages of development (Fig. 1). The Gout, Hyperuricemia and Crystal-Associated Disease Network (G-CAN) has previously endorsed a definition of the disease state ‘gout’ that requires current or prior clinically evident symptoms or signs resulting from MSU crystal deposition^16^. Gout is generally considered a chronic disease, with episodic highly symptomatic flares. Poorly controlled gout can have a substantial impact on an affected individual and the individual’s family. Inadequately treated gout leads to recurrent gout flares, the formation of tophi (which contain aggregated masses of MSU crystals in joints and certain soft tissues), chronic gouty arthritis and joint erosion. Ulceration and infection associated with tophi occurs frequently, and surgical interventions for these sequelae have a high rate of complications^17^. Substantial time off work, poor health-related quality of life and disability are common in those with poorly controlled gout^18–20^. Gout is associated with frequent hospital admissions, particularly in patients with hyperuricaemia and inadequate allopurinol use and/or dose^21,22^. However, not all individuals with gout develop severe disease, and whether everyone diagnosed with gout requires long-term urate-lowering therapy (ULT) has been questioned^23^.

**Fig. 2:**
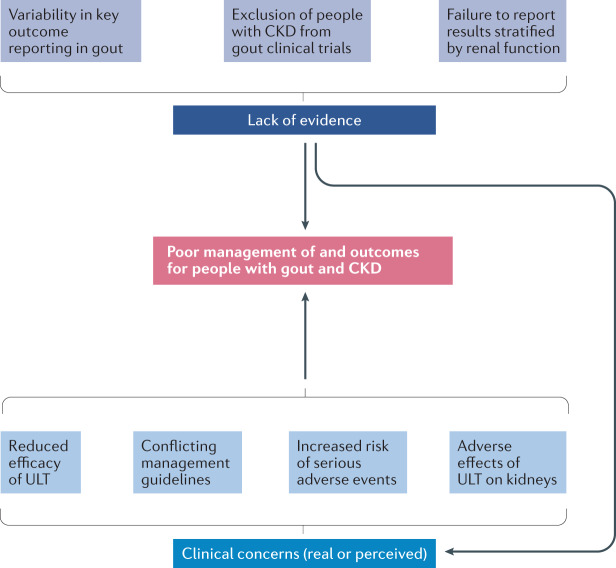
Reasons for poor outcomes in people with CKD and gout. This schematic provides a conceptual framework to explain poor management and outcomes in people with gout and chronic kidney disease (CKD). No good-quality evidence is available to guide treatment decisions because clinical trials have traditionally excluded participants with advanced CKD or, when these participants are enrolled, the trials have failed to report outcomes stratified by renal function. In addition, comparing and contrasting studies is difficult because of variability in reporting of outcomes for both urate-lowering therapy (ULT) and gout flare studies (this problem is not unique to gout in the context of CKD). In addition, many health-care team members involved in the management of people with gout and CKD have valid concerns about confusing guidance (conflicting recommendations among treatment guidelines from prominent societies), and harbour misconceptions (including that ULTs will have an adverse effect on renal function (and the ULT dose should therefore be adjusted), the risk of adverse effects (mainly allopurinol hypersensitivity) and that ULT will have reduced efficacy). These factors lead to excessively conservative approaches to the treatment of gout in people with CKD, which often does not achieve optimal treatment outcomes.

Care for people with gout and CKD presents important challenges. For instance, the clinical presentation of gout in this high-risk, comorbid population is variable, with a higher frequency of atypical presentations than in those without CKD^24^. However, quality evidence to guide the management of gout in people with CKD is lacking, owing at least in part to the exclusion of people with CKD from trials of gout therapies, failure to report results stratified by renal function and inconsistencies in the outcome measures used and reported^25,26^. The resultant knowledge gaps have contributed to concerns regarding gout treatment efficacy and safety, some of which are legitimate and others questionable^27,28^. The use of ULT in the context of advanced CKD varies greatly among rheumatologists, nephrologists and generalists^29^, and professional bodies have issued conflicting recommendations regarding the treatment of gout in people with concomitant CKD^27,28^. These inconsistencies frequently result in confusion and, consequently, suboptimal gout management with failure to achieve recommended target urate levels^30,31^. Moreover, pharmacological options for treating gout flares and lowering urate concentrations are often restricted by physicians, other health-care professionals (such as pharmacists) and patients who have appropriate concerns and/or misconceptions about drug toxicity or the need to adjust medication doses. As a result, outcomes in people with gout and CKD are commonly poor^32^ (Fig. 2).

This Consensus Statement from G-CAN aims to discuss the evidence (or lack of) for the management of gout in people with CKD and to identify key research questions that will address challenges faced in managing gout and CKD. We focus on CKD stages 3–5 (Table 1), for which there remain the most debate and concern about appropriate therapy for coexistent gout. This Consensus Statement is not intended as a guideline for the management of gout in CKD; rather, it analyses the available literature on the safety and efficacy of drugs used in gout management to identify important gaps in knowledge and associated areas for research. We do not analyse the role of ULT in people with asymptomatic hyperuricaemia and CKD, as treatment of asymptomatic hyperuricaemia with ULT is not currently recommended or approved in most areas of the world^28^. We do not consider non-pharmacological interventions for gout in CKD, such as dietary interventions or weight loss. Finally, this Consensus Statement does not discuss the particularly complex management and frequently severe clinical course of patients with hyperuricaemia and gout after transplantation of a kidney or other major organ.

## Methods

G-CAN comprises a group of individuals with expertise and an interest in gout and other crystal deposition-associated arthritic diseases as well as hyperuricaemia. G-CAN was formed to foster collaboration and research in these disease areas. During the G-CAN symposium in 2016, management of gout in the context of CKD was identified as an area of high interest, with critical gaps in knowledge about the efficacy and safety of drugs used for management of gout flares, gout flare prophylaxis and long-term ULT. G-CAN therefore endorsed systematic reviews of the evidence for the use of medications to manage gout flares as well as ULTs. These reviews^33,34^ form the basis for this Consensus Statement and provide the evidence to support key areas for research in each section. People with gout were not directly involved in this Consensus Statement. The work was led by C.L.H., L.K.S. and A.L.G. in collaboration with the G-CAN Directors (R.T. and H.K.C.) and Board. H.F., H.L.P., M.F. and A.B.V.-S. were selected as fellows with an interest in gout to assist with the work. The G-CAN Board, which includes the authors R.T., D.B.M. and H.K.C., approved the final manuscript.

### Literature review methods

As mentioned above, systematic literature reviews on the safety and efficacy of pharmacological therapies for gout in people with CKD were conducted to identify knowledge gaps and research priorities. The systematic literature review of ULTs^33^ was led by L.K.S. in conjunction with the fellows H.F. and A.B.V.-S., and the systematic literature review of therapies for gout flares and prophylaxis^34^ was led by A.L.G. and C.L.H. in conjunction with the fellows H.L.P. and M.F. We focused on medications currently approved for or commonly used for gout, including those used in the management of gout flares and flare prophylaxis when starting ULT (colchicine, NSAIDs, corticosteroids and IL-1 inhibitors) and those used as ULT (allopurinol, febuxostat, probenecid, benzbromarone, lesinurad and pegloticase).

Briefly, the search captured articles in PubMed, The Cochrane Library and EMBASE published from 1 January 1959 to 31 January 2018. Studies were included if they enrolled people with gout, an eGFR of <60 ml/min/1.73 m^2^ or a creatinine clearance of <60 ml/min and exposure to the medications of interest. Studies were excluded if they were not available in English, primarily included people without gout, did not report information on eGFR or creatinine clearance, were letters, opinion articles or review articles, or were animal studies, basic science or purely laboratory-based studies. For assessing efficacy of a ULT of interest, the main outcome was the proportion of study participants who achieved the target serum urate concentration of <6.0 mg/dl, stratified by renal function. Two reviewers independently screened the full texts to identify eligible studies for data extraction; any discrepancy identified during the screening phase was discussed by the two reviewers to reach consensus. Detailed methods and results of the literature reviews have been previously reported^33,34^.

### Identification of research areas

Data from the literature were thematically analysed by the leaders and fellows of each literature review team to identify general issues with the currently available data with regard to gout and gout studies in people with concomitant CKD as well as specific issues with individual medications.

The research areas and general requirements for studies identified were agreed on by the leaders and fellows of each literature review team and then circulated to all authors of this Consensus Statement. Agreement was reached by consensus of all authors via e-mail, and final approval was granted by the G-CAN Board. No effort was made to prioritize the research areas.

## Issues with studies of gout and CKD

Two important areas of concern were identified with respect to studies of gout and CKD: issues related to the natural history of gout in people with CKD and generic study-related issues.

### General research areas

Several issues related to the natural history of gout in people with CKD were identified as general areas for research (summarized in Box 1). As mentioned above, data on the natural history of gout (Fig. 1) are scarce but the application of modern imaging techniques, such as dual-energy CT, has led to the recognition of a pre-symptomatic phase of gout in some individuals in whom MSU crystal deposition occurs in joints, soft tissues and vascular sites before the first gout flare (asymptomatic MSU crystal deposition)^35,36^. Whether this pre-symptomatic phase is more common in people with CKD in whom the inflammatory response to crystals might be suppressed remains unknown, as does the timing of progression from asymptomatic to symptomatic gout in people with CKD.

Other questions to be addressed concern prediction of the disease course and the need for ULT in those with gout and CKD. The question of whether ULT is required could be an even more important issue in individuals potentially at increased risk of adverse events associated with therapy, such as those with CKD. An increased risk of mortality in people with gout, typically from cardiovascular and cerebrovascular disease, has been reported in association with the presence of subcutaneous tophi and high serum urate concentrations, but not with renal insufficiency^37^. Whether urate lowering in people with gout and CKD alters mortality was not considered as part of the literature reviews and this consensus statement.

Research is also needed to assess the effects of gout treatment on CKD and renal function. ULT in people with gout was reported in one study to lead to an improvement in renal function, although how much of this improvement related to a reduction in NSAID use and how much related specifically to the effects of urate lowering is not clear^38^. In addition, the results of this study were stratified only by baseline creatinine clearance of <80 ml/min versus ≥80 ml/min. A post hoc analysis of an allopurinol dose-escalation study suggested that changes in creatinine clearance did not differ when stratified by baseline renal function^39^. Stratifying the efficacy and safety of gout flare treatment and ULT by renal function should be emphasized in all gout studies because gout and CKD frequently coexist. An example of a trial designed to incorporate such stratification is the Veterans Affairs (VA) Stop GOUT study (NCT02579096), which is designed to evaluate the ‘treat-to-target’ dose escalation of allopurinol versus febuxostat in people with gout^40^. Although this study excluded individuals with stage 4 or 5 CKD, it included a pre-planned analysis of those with stage 3 CKD.

Box 1 G-CAN-proposed general research priorities for people with gout and CKD

- Is the natural history of gout, including the transition from asymptomatic hyperuricaemia to symptomatic gout, the same in people with and without chronic kidney disease (CKD)?
- In people with CKD and gout, can we predict who will develop tophaceous or erosive disease (and thus require more intensive urate-lowering therapy) and who will have a benign course?
- Does the treatment of gout and treat-to-target management of gout reduce progression of CKD and/or improve renal function?

G-CAN, Gout, Hyperuricemia and Crystal-Associated Disease Network.

### Study-related issues

The main generic study-related issues that contribute to a paucity of data on how to safely and effectively use medications for gout in people with CKD are discussed below, and G-CAN-proposed requirements for gout studies are summarized in Box 2.

Box 2 G-CAN-proposed requirements for pharmacological gout studies

- Whenever possible, people with all stages of chronic kidney disease (CKD) should be included in clinical trials of medications used in the management of gout.
- Pre-specified secondary analyses stratified by CKD stage should be reported for all clinical trials, cohort studies and observational studies.
- Pre-specified secondary analyses stratified by CKD stage for study participants with gout, independently of those with asymptomatic hyperuricaemia, should be reported for all clinical trials, cohort studies and observational studies.
- Standardized reporting of outcome measures, particularly serum urate concentrations and gout flares, are required to ensure that data can be compared across studies and meta-analyses can be undertaken.

G-CAN, Gout, Hyperuricemia and Crystal-Associated Disease Network.

#### Study populations

In general, people with substantial kidney impairment have been excluded from clinical trials, greatly limiting the data on which to base decisions regarding how to best treat gout in this population. Most pharmaceutical trials of newer therapies, such as febuxostat, excluded individuals with an eGFR of <30 ml/min/1.73 m^2^ (refs^41,42^) and in some cases excluded those with an eGFR of <60 ml/min/1.73 m^2^ (ref.^43^). Therefore, many data are derived from small case series, cohort studies and retrospective studies. Many studies with larger numbers of people with CKD included a mixture of those with asymptomatic hyperuricaemia and those with gout, but outcomes were not analysed separately.

#### Study reporting

Even when people with CKD were enrolled in clinical trials, few studies reported the outcomes stratified by kidney function. For example, only 12 of 96 articles reporting 91 original studies of allopurinol and 20 of 41 articles reporting 34 original studies with febuxostat in people with CKD and gout reported data according to renal function^33^. As a consequence, it is not possible to draw specific conclusions about the efficacy and safety of drugs used for gout in CKD. Furthermore, there are differences in the way outcome measures are reported. For example, serum urate was reported differently in these studies, as percentage reduction in serum urate, percentage of participants who achieved a target serum urate concentration, absolute reduction in serum urate and mean serum urate concentration at study end^25,44^. Likewise, gout flare was also inconsistently reported^25,26,34^. Such variability in study outcome reporting precludes meta-analysis, owing to difficulty comparing different studies.

## Drugs used for flares and prophylaxis

For drugs used in the management of gout flares or flare prophylaxis when starting ULT (namely NSAIDs, colchicine, corticosteroids and IL-1 inhibitors), the efficacy outcomes of interest are resolution or prevention of gout flares, respectively. For this G-CAN Consensus Statement, safety outcomes for each drug were individualized. The specific issues identified with medications used for the management of gout flares and prophylaxis in people with CKD are discussed below, and the G-CAN-proposed research priorities are outlined in Box 3.

Box 3 G-CAN-proposed research priorities for gout drugs in CKD **Colchicine**

***Treatment of gout flares***

- Safe and effective dosing of colchicine in chronic kidney disease (CKD): how should the AGREE trial^a^ colchicine dose be modified in different stages of CKD?
- How should colchicine be used in people with end-stage renal disease (ESRD) on dialysis?
- Is the risk of drug interactions with colchicine greater in patients with CKD?
- Whether the dose of colchicine should be altered when used in combination with atorvastatin in people with CKD.

***Gout flare prophylaxis***

- Can low-dose colchicine be used in people with ESRD on dialysis?
- Is there an increased risk of adverse effects with low-dose, longer-term colchicine use in people with CKD?

**NSAIDS**

***Treatment of gout flares***

- Are short-term NSAIDs safe in the context of ESRD?
- Are longer-term NSAIDs safe in the context of ESRD?

***Gout flare prophylaxis***

- Are some NSAIDs safer than others for longer-term prophylactic use?

**Glucocorticoids**

***Treatment of gout flares***

- What is the most appropriate duration of oral prednisone use for gout flares?

***Gout flare prophylaxis***

- Is there an increased risk of tophi in people receiving corticosteroids for gout flare prophylaxis?
- Is there a minimum safe dose or treatment duration in people in whom glucocorticoids need to be used for prophylaxis?

**IL-1 inhibitors**

***Treatment of gout flares***

- Is IL-1β inhibition a safe option in CKD?
- Are infection considerations of concern in people with gout?
- Should the dose of anakinra/canakinumab be adjusted based on kidney impairment?

***Gout flare prophylaxis***

- Is the use or dosing the same for flares as for gout flare prophylaxis?

**General**

***Gout flare prophylaxis***

- Is gout flare prophylaxis always required for people with gout and CKD starting urate-lowering therapy?

G-CAN, Gout, Hyperuricemia and Crystal-Associated Disease Network. ^a^The AGREE trial was a randomized, controlled trial of high-dose versus low-dose colchicine for managing gout flares^81^.

### Drugs used to manage gout flares

NSAIDs are generally contra-indicated in people with CKD, and the published literature in gout generally aimed to show the potential for renal-related adverse effects in people with CKD^34^. Although NSAIDs have well-established adverse effects, there has been some suggestion that these drugs could be used in those with end-stage renal disease for short periods of time^45^.

There are a small number of randomized controlled trials (RCTs) of colchicine for treatment of gout flares, and none of these reported outcomes stratified by renal function^34^. Pharmacokinetic studies have indicated that clearance of colchicine is decreased in those with severe kidney impairment (eGFR 15–29 ml/min/1.73 m^2^) and that there is minimal clearance of colchicine by haemodialysis^46^. Thus, the recommendations for use of colchicine in CKD remain largely empirical.

Corticosteroids have been generally accepted as the safest option in most people with gout flares and concomitant CKD. The newer IL-1 antagonist therapies, such as canakinumab and anakinra, are not widely available, and there are no RCTs investigating their use in people with gout and CKD for which results are presented according to kidney function. Data from case series and case reports are reassuring^34^. Essentially, these agents are widely used for a variety of conditions, but there is a relative paucity of data on their use in people with gout and CKD. In people without gout, the clearance of anakinra has been shown to be directly related to renal function and the drug is not cleared by dialysis^47^. It has therefore been suggested that in patients with an eGFR of <30 ml/min/1.73 m^2^, anakinra should be administered every other day^47^. By comparison, canakinumab is a human IgG with a large molecular size (∼150 kDa), so not much renal excretion is expected^48^.

### Drugs used for flare prophylaxis

Although the medications used for flare prophylaxis are the same as those used to treat flares, they are generally used at lower doses and for longer periods of time (months rather than days or weeks). A post hoc analysis of three phase III RCTs in people starting febuxostat who also received prophylaxis with colchicine included participants with an eGFR of <30 ml/min/1.73 m^2^ but again the results were not stratified by renal function^49^. Long-term use of colchicine in the general population has been associated with bone marrow suppression and neuromyotoxicity^50^, but whether these effects are increased in those with gout and CKD is unknown. Whereas short-term courses of glucocorticoids can be considered to have an acceptable risk–benefit profile, long-term use of glucocorticoids for flare prophylaxis can be associated with an increased risk of glucocorticoid-related adverse events, particularly infections, as seen in other rheumatic diseases^51,52^. This risk could be particularly concerning in a population that is already at high risk of severe infections, such as those with CKD. Whether the gout flare rate when starting ULT is the same in those with CKD as in those without, and whether prophylaxis is always required, are unknown, although a recent study of incremental use of febuxostat suggested that prophylaxis might not be required when a dose-escalation approach is used^53^.

## Urate-lowering therapies

The appropriate use of ULT in people with gout and CKD is one of the most controversial areas of gout management. For example, the latest guidelines issued by the ACR, EULAR and the British Society for Rheumatology differ in important areas, such as allopurinol dosing in people with CKD^27,28,54^. For drugs used as ULT (allopurinol, febuxostat, probenecid, benzbromarone, lesinurad and pegloticase), efficacy outcomes, as endorsed by most rheumatology professional society management guidelines, include achieving a target serum urate concentration (that is, <6 mg/dl or <5 mg/dl), resolution of tophi, reduction or elimination of gout flares over time, improvement in quality of life indicators, and radiographic changes^27,28,54^. For this G-CAN Consensus Statement, safety outcomes for each drug were individualized. The general issues identified as well as specific issues with individual drugs are discussed below and the G-CAN proposed research priorities are outlined in Box 4.

Box 4 G-CAN-proposed research priorities for ULT in CKD **Allopurinol**

- Does commencing allopurinol at a lower dose reduce the risk of allopurinol hypersensitivity syndrome (AHS)?
- How quickly can allopurinol dose be escalated while avoiding AHS and/or severe cutaneous adverse drug reactions?
- Which are the most important risk factors for AHS, and can we more accurately predict who will get AHS-based risk factors?
- Can dialysis improve outcomes in people with AHS?
- Does starting allopurinol at a low dose and gradually increasing the dose reduce the risk of flares and thus alleviate the need for flare prophylaxis?
- Can we predict the dose of allopurinol required to achieve the target urate concentration?
- Does allopurinol provide protection for the heart or kidneys in people with gout, chronic kidney disease (CKD) and cardiovascular disease?
- Is there a differential between peritoneal and haemodialysis with regard to urate lowering?

**Febuxostat**

- Is febuxostat neutral or associated with an increased risk of cardiovascular death in people with gout, CKD and cardiovascular disease?
- Is febuxostat safer than allopurinol in CKD?
- Are lower starting doses of febuxostat (10–20 mg) less likely than higher doses to cause flares in those who have no good options for prophylaxis?

**Probenecid**

- At what level of kidney function is probenecid ineffective?
- Is combination therapy with xanthine oxidase inhibitors safer or more effective than probenecid monotherapy?

**Benzbromarone**

- Is the risk of hepatotoxicity lower in those receiving benzbromarone 100 mg daily compared with higher doses?
- Is there a level of CKD at which benzbromarone should not be used?

**Pegloticase**

- Is there any difference in risk of immunogenicity with pegloticase in CKD?
- What is the role of concomitant immunosuppression to avoid anti-drug antibodies in those with CKD?
- Does CKD alter the indications for debulking of palpable and erosive tophaceous disease in CKD using recombinant PEGylate uricase therapy?
- Is earlier tophaceous disease debulking in CKD, using recombinant PEGylate uricase therapy, a better approach than initial conventional oral urate-lowering therapy (ULT)?
- Is the effect of rebound flares as severe in advanced CKD?

G-CAN, Gout, Hyperuricemia and Crystal-Associated Disease Network.

### Level of renal function precluding ULT

Because most large RCTs have excluded people with substantial renal impairment, there are few data from RCTs to inform decisions about when specific ULTs should not be used on the basis of kidney function. No studies have specifically examined the risks and benefits of not treating gout in people with CKD with ULT, and all current guidelines recommend ULT treatment in this population. In many patients, but not all, untreated gout causes considerable morbidity in its own right, and in those with CKD the only option for treating flares might be long-term corticosteroids, which is associated with further morbidity.

In general, two main reasons are given for avoiding ULT in people with CKD: lack of efficacy and an increased risk of adverse events. There is a general reluctance to use allopurinol in those with an eGFR of <30 ml/min/1.73 m^2^ owing to concerns about the risk of allopurinol hypersensitivity syndrome (AHS) and poor outcomes in those with substantial renal impairment who develop AHS^55^. Despite fewer data for febuxostat than for allopurinol, there has been more acceptance of using febuxostat in people with CKD, on the basis of the knowledge that febuxostat is mainly metabolized in the liver and is not dependent on renal function for excretion. One popular school of thinking is that probenecid is ineffective in patients with an eGFR of <50 ml/min/1.73 m^2^ and therefore should generally be avoided in this setting, but data suggest otherwise^56^. Benzbromarone is effective even in those with eGFR as low as 20 ml/min/1.73 m^2^, but it is not available in many countries owing to the risk of hepatotoxicity. Lesinurad was rapidly determined to be contraindicated in those with an eGFR of <30 ml/min/1.73 m^2^, given an increased risk of worsening kidney function, and the drug is no longer marketed. Whether the combination of xanthine oxidase inhibitors (XOIs; allopurinol or febuxostat) with uricosurics (such as probenecid) is a viable strategy in people with gout and CKD is unknown as these combinations share the same limitations of uricosurics by themselves, and evidence that is even more limited. Pegloticase is largely under-studied although the available data suggest it has similar efficacy and safety in those with impaired kidney function and those with normal kidney function^33^. As there are some data indicating that the frequency of gout flares decreases with advancing CKD and after dialysis, it is plausible that some patients with mild hyperuricaemia or normouricaemia and no flares will not require ULT^57^.

### ULT with renal replacement therapy

There is a paucity of data on the safety and efficacy of ULT in people on haemodialysis, and even less in those on peritoneal dialysis. It has been suggested that haemodialysis should reduce serum urate concentration such that specific ULT is no longer required^58,59^. However, this is not a universal finding^60^. It has also been reported that serum urate is at the target concentration less often in those on haemodialysis than in those on peritoneal dialysis, perhaps due to the intermittent rather than continuous removal of urate through dialysis^60^. The data for use of allopurinol and febuxostat in patients undergoing haemodialysis are predominantly limited to case reports and case series^61–65^. For allopurinol, detailed information about the effect of haemodialysis on plasma concentrations of oxypurinol (the active metabolite of allopurinol) indicates that it is effectively dialysed^66^ and suggests that allopurinol should be given after haemodialysis.

### Appropriate dosing of XOIs

As mentioned above, allopurinol dosing in CKD is one of the most controversial areas in gout management owing to the risk of AHS in people with CKD. On the basis of primarily case series and a retrospective case–control study^67^, there is general agreement that the starting dose of allopurinol should be low and increased slowly, although no prospective trial data are available to prove or disprove the rationale that such an approach will reduce the risk of AHS. Use of allopurinol is further complicated by the large inter-individual variability in the dose required to achieve the target serum urate concentration (100–900 mg daily). Despite data suggesting that allopurinol dose escalation can achieve target serum urate concentrations even in those with kidney impairment^39,68,69^, the belief that the allopurinol dose should be reduced in people with CKD (‘renally dosed’) remains pervasive. In comparison to allopurinol, febuxostat has a narrower dose range (40–120 mg daily) and there has been more willingness to use febuxostat in people with CKD. In the largest study of febuxostat in CKD, which enrolled 96 people with an eGFR in the range 15–50 ml/min/1.73 m^2^, febuxostat 60–80 mg daily was associated with a reduction in serum urate concentration (compared with placebo) with no decline in renal function^70^.

### Hypersensitivity reactions to XOIs

Both allopurinol and febuxostat have been associated with hypersensitivity reactions, which can be severe with either drug^71,72^. For allopurinol-related reactions, a number of risk factors in addition to kidney impairment have been identified, including the allopurinol starting dose, the presence of HLA-B*58:01 and concomitant use of diuretics^73^. The interaction between the identified risk factors, particularly allopurinol starting dose, renal impairment and HLA-B*58:01, seems to be especially important. As might be expected with any life-threatening reaction, mortality is higher in those with pre-existing CKD^55^. Oxypurinol concentration, which is influenced by allopurinol dose, the presence of diuretics and renal function, might have a role in the pathophysiology of AHS. In vitro studies have shown allopurinol hypersensitivity to be mediated by an oxypurinol-specific T cell response, and drug concentration is an important factor in T cell sensitization^74,75^. However, there is no evidence that a specific oxypurinol concentration precipitates AHS, as many individuals tolerate high concentrations and AHS has been reported in some with low concentrations, indicating that other factors must be involved. The critical combination of risk factors in HLA-B*58:01-negative individuals remains to be determined. Currently, treatment of AHS is supportive. The combined evidence that those with CKD and high oxypurinol concentrations have a poorer outcome and that oxypurinol is readily dialysed begs the question as to whether early dialysis can improve outcomes in people with AHS. There are no data about CKD or renal function and the risk of drug reaction with eosinophilia and systemic symptoms (DRESS) associated with febuxostat treatment.

### Cardiovascular risk with XOIs

In the general population, CKD is known to be associated with an increased risk of cardiovascular disease (CVD)^76^. There has been debate about the use of febuxostat in people with CVD given the results of the Cardiovascular Safety of Febuxostat and Allopurinol in Patients with Gout and Cardiovascular Morbidities (CARES) trial^77^ and the Febuxostat versus Allopurinol Streamlined Trial (FAST)^78^. CARES was a large RCT in people with gout and pre-existing CVD conducted in the USA that found no increased risk related to treatment with febuxostat compared with allopurinol for the primary end point, which was a composite of cardiovascular death, non-fatal myocardial infarction, non-fatal stroke or unstable angina with urgent revascularization (HR 1.03, 95% CI 0.87–1.23). People with an eGFR of <30 ml/min/1.73 m^2^ were excluded from the study but the risk of these events did not differ in those with normal, mild or moderate kidney impairment. However, pre-specified secondary analyses revealed an increased risk of cardiovascular-related death (HR 1.34, 95% CI 1.03–1.73) and death from any cause (HR 1.22, 95% CI 1.01–1.47) in those receiving febuxostat compared with allopurinol; unfortunately, there was no stratification by renal function in the secondary analysis^77^. Although there are a number of issues with the CARES study^79^, it raised issues about the relative safety of allopurinol and febuxostat in people with gout and CVD and led to a black box warning for febuxostat use. The results of the CARES study have been challenged by FAST, another large RCT conducted in European countries, which also compared the cardiovascular safety of febuxostat and allopurinol in patients with gout^78^. Enrollees in FAST had at least one additional cardiovascular risk factor, but patients with advanced CKD were excluded. Febuxostat was non-inferior to allopurinol for the primary end point (a composite of hospitalization for non-fatal myocardial infarction or biomarker-positive acute coronary syndrome; non-fatal stroke; or cardiovascular death). In contrast to the CARES trial, FAST found that treatment with febuxostat was not associated with an increase in cardiovascular death or all-cause death. Overall, fewer deaths occurred in the febuxostat group than in the allopurinol group^78^. When comparing the CARES trial and FAST, FAST had more complete follow-up and better event adjudication (linked to national databases), which provides reassurance about the use of febuxostat, although the findings cannot be directly extrapolated to patients with advanced CKD. Whether CKD modulates this risk or whether febuxostat has a better cardiovascular safety profile than allopurinol in this population remains to be determined.

### Role of combination ULT

Combination therapy with a XOI and a uricosuric can be very effective, and if uricosuric toxicity is a consequence of urate concentration within renal tubules then combination therapy could theoretically ameliorate such toxicity. However, as uricosuric treatment is usually not considered for patients with advanced CKD this approach is largely untested.

## Conclusions

This Consensus Statement highlights where knowledge regarding the management of gout in people with CKD remains incomplete, and proposes a research agenda to address the most important areas of uncertainty, which includes a better understanding of the natural history of gout in people with CKD. A greater knowledge of the safety of treatments used for the management of gout flares, as well as the requirement for flare prophylaxis in this population, is also needed. Additional investigation is required to determine the safe dosing of allopurinol in people with CKD, as well the prediction and management of AHS. Further research is also required to help determine whether febuxostat is associated with increased cardiovascular risk or is in fact risk-neutral, and whether febuxostat is safer to use than allopurinol in people with gout and CKD. The safety and efficacy of uricosuric medications at different levels of renal function is another area where further research would be of benefit. Evidence regarding the use of pegloticase in people with gout and CKD is limited. From the standpoint of treating or preventing gout flares, the knowledge gaps are also substantial and revolve around the safe use and dosing of colchicine, the safety and efficacy of IL-1 inhibitors and the absolute indication for prophylactic therapy in all patients in whom ULT is being initiated.

In order to resolve these issues, it is important that researchers include patients with all stages of CKD in clinical trials of gout management wherever possible, and undertake pre-specified analyses of safety and efficacy according to renal function, using standardized outcome measures.
